# Risk inventory and mitigation actions for AI in medical imaging—a qualitative study of implementing standalone AI for screening mammography

**DOI:** 10.1186/s12913-025-13176-9

**Published:** 2025-07-30

**Authors:** Annika Gerigoorian, Maha Kloub, Karin Dembrower, Mats Engwall, Fredrik Strand

**Affiliations:** 1 Department of Industrial Economics and Management, KTH Royal Institute of Technology, Stockholm, Sweden; 2 Department of Oncology-Pathology, Karolinska Institutet, Anna Steckséns gata 30A, Stockholm, SE 171 64 Sweden; 3 Department of Breast Radiology, Capio S:t Göran Hospital, Stockholm, Sweden

**Keywords:** Artificial Intelligence, AI Integration, Enterprise Risk Management, Mammography, Medical Imaging

## Abstract

**Background:**

Recent prospective studies have shown that AI may be integrated in double-reader settings to increase cancer detection. The ScreenTrustCAD study was conducted at the breast radiology department at the Capio S:t Göran Hospital where AI is now implemented in clinical practice. This study reports on how the hospital prepared by exploring risks from an enterprise risk management perspective, i.e., applying a holistic and proactive perspective, and developed risk mitigation actions.

**Method:**

The study was conducted as an integral part of the preparations before implementing AI in a breast imaging department. Collaborative ideation sessions were conducted with personnel at the hospital, either directly or indirectly involved with AI, to identify risks. Two external experts with competencies in cybersecurity, machine learning, and the ethical aspects of AI, were interviewed as a complement. The risks identified were analyzed according to an Enterprise Risk Management framework, adopted for healthcare, that assumes risks to be emerging from eight different domains. Finally, appropriate risk mitigation actions were identified and discussed.

**Findings:**

Twenty-three risks were identified covering seven of eight risk domains, in turn generating 51 suggested risk mitigation actions. Not only does the study indicate the emergence of patient safety risks, but it also shows that there are operational, strategic, financial, human capital, legal, and technological risks. The risks with most suggested mitigation actions were ‘Radiographers unable to answer difficult questions from patients’, ‘Increased risk that patient-reported symptoms are missed by the single radiologist’, ‘Increased pressure on the single reader knowing they are the only radiologist to catch a mistake by AI’, and ‘The performance of the AI algorithm might deteriorate’.

**Conclusion:**

Before a clinical integration of AI, hospitals should expand, identify, and address risks beyond immediate patient safety by applying comprehensive and proactive risk management.

**Supplementary Information:**

The online version contains supplementary material available at 10.1186/s12913-025-13176-9.

## Background

The clinical uptake of AI has been relatively slow and there is a paucity of studies that focus on the risks and risk mitigation actions when replacing physicians with AI. A study carried out by Lång et al. (2023) [1] demonstrated that AI-supported mammography screening yielded a comparable cancer detection rate to traditional double reading, significantly reducing the workload for radiologists. Furthermore, results from the ScreenTrustCAD trial conducted at Capio S:t Göran Hospital in Sweden aimed at evaluating the performance of an AI algorithm for mammography screening revealed that one radiologist working in parallel with an AI algorithm detects more cases of breast cancer than the traditional procedure with two radiologists, whilst recalling fewer healthy women [2]. Subsequently, the hospital implemented AI into their mammography screening workflow to replace one of two radiologists.

Successful AI integration in healthcare necessitates organizational readiness and workflow adaptability [3]. However, the diverse characteristics of healthcare organizations can slow down their responsiveness to change [4]. The involvement of numerous stakeholders, adherence to standardized procedures, and regulatory requirements further complicate the integration process [3]. Incorporating AI for diagnostics introduces uncertainties concerning ethics, law, and competence [5], especially when AI is used as a decision-maker [6].

Traditional risk management programs in hospitals primarily focus on reactive responses to incidents, aiming to ensure patient safety [7]. However, with the increasing prevalence of AI in healthcare, a more proactive approach encompassing a broader perspective on risks and risk mitigation is necessary. The Enterprise Risk Management (ERM) components, governance and culture, strategy and objective-setting, performance, review and revision, and information, communication, and reporting [9]. ERM is however not yet fully established in healthcare settings [8], and only a few studies discuss the framework from a healthcare perspective or utilize it as a basis for risk inventory when introducing new technologies within healthcare settings. This study applies an ERM-inspired framework to broaden the identification of risk and mitigating actions beyond patient safety associated with AI implementation in screening—a perspective not commonly addressed in existing literature.

## Objectives

The main objective of this study was to use ERM-inspired domains to broaden the perspective when performing a risk inventory in for a before integrating AI for image interpretation, particularly when considering the replacement of a physician with AI. This paper identifies potential risks and proposes a strategy for mitigating these risks from the standpoint of enterprise risk management. The goal of this research is to offer practical and applicable findings that ought to be integrated into the preparations for AI integrations within healthcare settings.

## Methods

### Empirical setting, research design, analytical framework

The study was designed as a qualitative single case study, focusing on Swedish Capio S:t Göran as the first hospital to clinically implement AI as a replacement for the second reader in population-wide mammography screening. Participants employed at Capio S:t Göran Hospital, selected based on their involvement in the AI integration, took part in two group sessions with three participants each as well as four sessions with one participant (See Table 1), ensuring diverse perspectives.

**Table 1:** Overview of the collaborative ideation sessions for the first step—inventory of risks

Participants	Number of participants	Format	Duration
Breast radiologist	3	In-person	30 min
Controller	1	In-person	30 min
IT manager	1	In-person	40 min
Operations Manager	1	In-person	30 min
Radiographer	3	In-person	30 min
Responsible person for another AI project	1	In-person	30 min

**Fig. 1:**
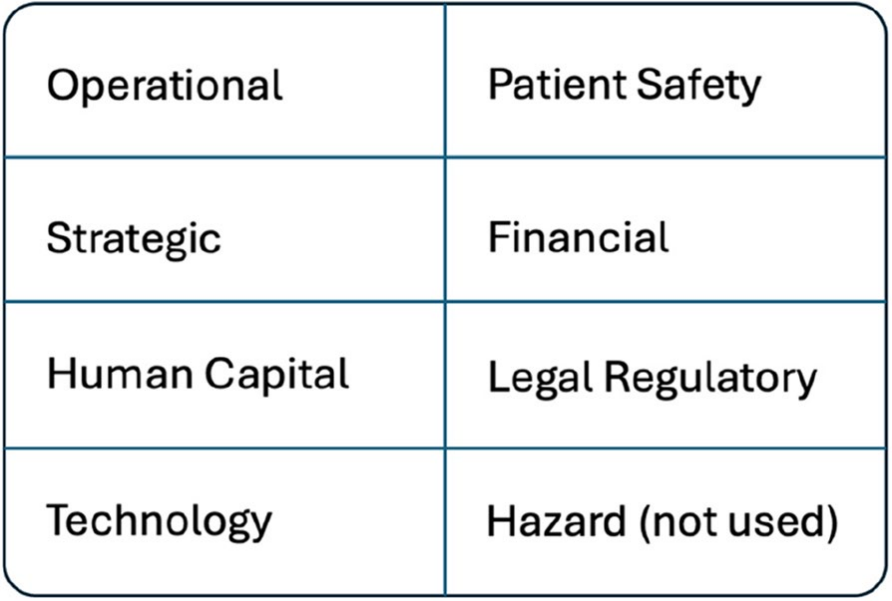
The eight risk domains from the ERM framework adapted by the American Society for Healthcare Risk Management

The Enterprise Risk Management (ERM) framework proposed by the American Society for Healthcare Risk Management guided the methodological approach of our study [9].The eight risk domains from this framework are shown in Fig. 1: This framework is structured in eight risk domains: Operational, Patient Safety, Strategic, Financial, Human Capital, Legal/Regulatory, Technology, and Hazard.

**Fig. 2:**
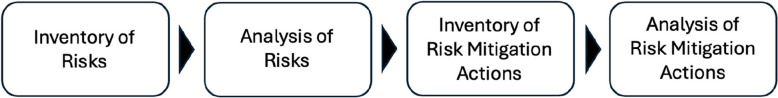
The risk management research process

To produce a collection of risk mitigation actions, we followed a research workflow as described in Fig. 2. The steps were inventory of risks, analysis of risks, inventory of risk mitigation actions, and analysis of risk mitigation actions. Risks were structured using ERM domains, but we did not formally apply ISO 31000 or other standardized risk analysis processes.

Risk and mitigation actions inventory were determined through a series of collaborative ideation sessions (also known as brainstorming), in-depth interviews, and joint analysis sessions, all of which were moderated by two non-healthcare professionals (AG, MK) by using the framework provided to conduct semi-structured interviews. The specific process for each step is detailed in the four sections below. Beyond the framework, more detailed questions were not predefined. The moderators ensured that all eight domains were covered in all sessions with the participants. The moderators used prompts such as'What could go wrong if AI replaced the radiologist?'and'Which aspects of implementation are most uncertain?'.

### Inventory of risks

Risks associated with AI in screening mammography were identified through collaborative ideation sessions involving hospital staff and experts led by two co-authors (AG, MK) who were engineering master students without prior involvement in the AI integration work at the hospital. These sessions allowed for open discussions and comprehensive exploration of risks, including those that may not surface in regular interviews [9]. The moderators introduced the eight risk domains, and participants considered the impact of AI on patients, radiologists, staff, and the organization while identifying risks and uncertainties. When conducting the group collaborative ideation sessions, the participants were first asked to write down their thoughts to prevent any influence from others. Subsequently, an open discussion was held, allowing the group to share their notes and further discuss the identified risks. During the individual sessions, participants were asked the same questions as in the group sessions but were encouraged to express their thoughts and concerns orally without providing written responses.

To complement with additional external perspectives, we conducted 5 semi-structured interviews, each lasting approximately 30 min. Participants were asked open-ended questions prompted by the eight enterprise risk management domains, including topics such as workflow disruption, liability, and technological integration. The semi-structured interviews were conducted with two external experts at KTH Royal Institute of Technology; one Ph.D. student in cybersecurity and machine learning, who provided insights into security risks and technical aspects of AI, and one Associate Professor in human–computer interaction, who offered insights into the social and ethical implications of AI in healthcare. At the start of the interview, they received an oral description of the studied AI integration case but were not informed about any information collected from the internal participants.

### Analysis of risks

Based on the information collected during the collective ideation sessions and interviews described above, a thematic data analysis was performed by three co-authors (AG, MK, FS) to structure the information. Concerns and risks were extracted from the interview notes and translated from Swedish to English. Each specific risk was labeled with a descriptive code and linked to one of the eight risk domains proposed by the ERM framework, and, if applicable, to a second domain on which it was considered to have an indirect impact.

### Inventory of risk mitigation actions

Two co-authors (AG, MK) presented the identified specific risks and the impacted risk domains. A collective ideation session was conducted, involving the co-author and principal investigator (FS), the co-author and head physician of the breast imaging department (KD), and the IT manager for radiology and functional imaging. After the presentation of each specific risk, the two co-authors (AG, MK) encouraged discussion and collected proposed mitigation actions. While the IT manager primarily focused on risks related to Technology and Operations, the head physician and research leader contributed insights across all domains.

### Analysis of risk mitigation actions

After the ideation session was concluded, the same participants participated in an assessment where the effectiveness and suitability of each mitigation action in addressing the identified risks were discussed. Only risk mitigation actions for which there was consensus that they would be effective and possible to implement, remained in the final risk mitigation strategy. Actions were grouped into themes.

## Results

### Risk inventory

In total, 87 risks were identified during the collaborative ideation sessions and expert interviews. After reviewing the relevancy of the risks, refining them, and reducing redundancies, 23 risks remained [see Table S1 in the Additional file 1]. Worth noting is that risks were identified within seven of the eight risk domains of the ERM framework, but not in the hazard domain. In the following sections, the identified risks within the seven risk domains are described.

### Operational risks

Three operational risks were identified. The introduction of AI and the potential replacement of a physician with an AI system may increase patient inquiries and disrupt the internal workflow, impacting workflow efficiency but also patient satisfaction. Workflow disruptions and delays could furthermore arise from IT-support deficiencies, AI system shutdowns, or the AI system reaching its technical end-of-life. These scenarios could indirectly affect patient safety by leading to delayed diagnoses. Lastly, the IT department’s ability to maintain continuous AI availability was identified as a risk. Delays in AI availability could significantly impact the intended workflow, potentially leading to failed internal processes.

### Patient safety risks

Four primary risks were identified in the Patient Safety domain. All participants emphasized the criticality of patient safety during AI integration. One significant risk, highlighted by both radiologists and radiographers, was the potential oversight of radiographer notes of patient symptoms when having a single radiologist. This risk has implications not only for patient safety but also for the operational domain, potentially impairing proper screening, and may also increase the possibility of legal claims.

Another issue was the risk of setting the AI threshold value too high, or too low. This threshold value determines which women that are to be flagged for further assessment in the consensus discussion and if the radiologist agrees, which women are to receive a letter informing them that no cancer signs were found. A high threshold could increase the likelihood of false negatives, potentially missing cancer, and compromising patient safety. In contrast, setting the threshold too low may lead to an excessive number of false positives, resulting in unnecessary follow-up procedures affecting operational processes.

Furthermore, respondents highlighted the risk of automation bias, wherein radiologists may excessively rely on AI decisions and overlook their judgment. Finally, the unknown performance of AI in unique cases, such as women with breast implants, was another concern.

### Strategic risks

The integration of AI in the hospital setting presented several strategic risks. Patients with a lack of understanding of AI systems, coupled with higher expectations of machines’ accuracy and performance compared to those of human professionals, could result in patient dissatisfaction and a loss of trust.

In addition, the reliance on a third-party AI vendor introduces vulnerabilities related to data privacy and security. Additionally, the hospital’s strong dependence on the AI vendor may lead to lock-in effects, where switching to an alternative vendor would become costly and challenging.

### Financial risks

Long-term price development of the AI system was one of two financial uncertainties highlighted by the participants. There is a risk that the cost of the system may increase over time, which could create a financial burden for the hospital. This uncertainty could disrupt strategic plans that rely on stable and predictable partnerships with AI vendors.

Additionally, the limited lifespan of technology introduces the risk of repeated investments in the future. As technology evolves and advances, there is a possibility that the AI system implemented may become obsolete, or requires upgrades, necessitating further financial commitments.

### Human capital risks

Four human capital-related risks associated with the integration of AI were identified. One prominent risk highlighted by the participants was the deilling of future radiologists. The concern stemmed from whether junior radiologists would be able to acquire the same level of screening competence and experience as their senior colleagues, given that AI would essentially remove the need for a second radiologist.

Another risk identified was the lack of understanding among radiographers and radiologists regarding the integration of AI. The participants expressed concerns about the limited knowledge of AI’s performance and integration. The radiographers desired greater involvement in the integration processes to overcome the sense of exclusion.

The increased pressure imposed on the first reader radiologist when AI replaces the second reader was also a risk identified. Single radiologists may experience heightened stress and pressure, knowing that they are solely responsible for catching any mistakes by AI.

The risk of personnel not accepting the AI integration was identified as a human capital risk common in the initial stages of the integration. The participants did however observe that these concerns diminished over time as they gained firsthand experience with AI and realized its benefits.

### Legal/regulatory risks

Legal and regulatory risks associated with the integration of AI were identified with the Legal/Regulatory Risks domain ranked as the second highest in terms of criticality, following the Patient Safety domain.

One of the primary legal risks identified was the uncertainty surrounding current and upcoming laws and regulations. Compliance with various complex regulations, such as the patient data act, Medical Device Regulation (MDR), General Data Protection Regulation (GDPR), CE mark, and the upcoming AI Act, poses challenges for the AI system.

Another significant concern raised by nearly all respondents pertained to the allocation of responsibility for mistakes or misconduct resulting from the introduction of AI in clinical settings and the replacement of a radiologist with AI.

### Technology risks

One significant risk discussed by the participants was the risk of AI malfunctioning due to system errors or bugs. Participants also highlighted concerns related to future updates of the algorithm and their impact on its performance. Replacing the current mammography equipment was noted as an additional factor affecting the algorithm’s performance.

Another identified risk pertained to cybersecurity concerns associated with the use of AI and even more so with IT services in the cloud. However, Capio S:t Göran Hospital plans to use an on-premises installation.

The study participants also discussed the risk of any AI system’s output being difficult for radiologists to interpret, which could impact the delivery of care, particularly for new radiologists.

### Identification of risk mitigation actions

#### Internal communication measures

One of the most significant risk mitigation themes identified pertains to the significance of implementing effective internal communication measures. The results suggest that information sharing plays a vital role to facilitate patient’s understanding of the AI integration, encompassing mitigation actions such as collaborative ideation potential patient questions and responses. Furthermore, displaying information regarding the use of AI in waiting rooms can address patient concerns. Promoting clear and consistent communication among stakeholders is also essential, with regular workplace meetings to monitor radiographer and radiologist experiences and address patient concerns. These actions are also crucial to alleviate the increased pressure on single readers responsible for catching mistakes made by the AI. Communicating the effectiveness of AI through internal evidence and published studies helps build trust and alleviate concerns about missed findings. In addition, to mitigate the risk of patient-reported abnormalities being missed when AI replaces a radiologist, the radiographers will be asked to set a workflow flag in the hospital’s Picture Archiving and Communication System (PACS) which would steer those cases to a special list.

### Diagnostic quality assurance measures

The findings showed that diagnostic quality assurance measures are crucial in mitigating operational and clinical patient safety risks associated with the integration of AI. This was reflected in several of the identified risk themes, particularly those related to staff retraining, liability, and reliance on vendor support. These measures focus on continuously ensuring the accuracy and reliability of AI-based outcomes. There should be a regular follow-up to detect systematic changes in the distribution of AI scores, overall and for the subgroups of (1) exams flagged by the radiologist, (2) exams recalled by the consensus discussion, and (3) exams followed by cancer diagnosis within a specific follow-up time. For validating new versions of the AI system, a potential mitigation measure may be to use the Swedish VAI-B platform, onto which images from a hospital can be uploaded and tested against a panel of commercial AI systems [10]. While initial results have been positive, scientifically validating the AI’s breast detection performance in cases with implants and other unique scenarios was deemed necessary by respondents.

### Educational measures

Knowledge sharing and letting junior radiologists make shadow assessments could facilitate skill development and decrease the risk of junior radiologists not acquiring the same screening competence and experience as their senior colleagues. Designing comprehensive training programs and evaluation systems for radiologists was also identified as a vital measure.

### External communication

The results showed that it is crucial to go beyond individual patient interactions and engage in broader discussions to raise awareness and address concerns. This involves collaborating on a national and European level to establish guidelines and standards, manage the liability concerns with AI, and receive clarity on calibrating the AI threshold value.

### Vendor management

Vendor management was also identified as a crucial aspect of risk mitigation when implementing AI in mammography. This also includes being prepared for potential changes to a different AI provider in the future.

### Legal assistance

When implementing AI, the legal and regulatory implications must be understood. To address legal uncertainties, consulting legal experts, clarifying responsibilities, and complying with laws and regulations is crucial.

### IT security compliance

To deal with cybersecurity issues that may arise with the use of AI, it was recommended to conduct a specific evaluation of potential security risks introduced by AI and accordingly implement robust security protocols at the hospital.

## Discussion

This study outlines potential risks with the clinical integration of AI for screening purposes and the replacement of a physician with AI. We applied the ERM framework to analyze risks related to AI implementation. Unlike traditional risk management, which often focuses on safety, ERM encompasses strategic, operational, and reputational domains. It also suggests measures for how to manage and mitigate the identified risks. As the ERM framework of ASHRM postulates there exist risks beyond patient safety risks, which our study demonstrates [9]. Since the findings indicated risks mapped to seven of the eight domains of the ERM framework, it strengthens the rationale for hospitals to adopt a risk management approach that considers multiple categories of risk.

It is important to highlight that risks are interdependent and that various risk domains may impact each other. This study demonstrated that 21 of the 23 risks identified have indirect impacts on other domains. As an example, it was apparent that several risks occurring and affecting a domain other than the patient safety domain could still impact patient safety. Despite that an enterprise risk management approach emphasizes a comprehensive view of risks where not only patient safety risks should be in focus, the delivery of high-quality and safe patient care must be the primary objective of healthcare organizations [8, 11, 12]. Being aware of secondary risk impacts is thus crucial. For instance, the risk that the AI algorithm’s performance deteriorates due to system updates, or a change of radiology equipment, might initially be considered as a technological issue, but ultimately it might jeopardize the delivery of safe care. This corroborates the ERM’s idea of maintaining a comprehensive risk management approach to not only identify risks from different dimensions but also to illuminate how the risks are interrelated.

Previous research has highlighted liability uncertainties associated with AI’s role in instances of patient care misconduct [6, 13–15]. This paper contributes by applying enterprise risk management to identify and organize real-world risks associated with AI implementation in mammography screening. There is currently a lack of regulations and trial procedures that specifically address the issue of accountability in cases AI fails to detect cancer [12, 14, 15]. This advocates the need to increase awareness for clarifying the ambiguities surrounding liability at both the national and European levels.

Previous research has also shown the significance of algorithm validation for safe AI adoption in hospitals [10, 16]. In line with this, our findings emphasize that performance deterioration over time represents a critical risk — highlighting the need not only for initial validation but for continuous monitoring and revalidation throughout the algorithm’s lifecycle. Although an AI system performs well in the current setting, uncertainties remain about maintaining diagnostic accuracy with AI model updates, changes in radiology equipment, or changes in the patient population.

Furthermore, concerns regarding AI resulting in deskilling have also been addressed in previous research [17–20]. As noted here, AI replacing the role of the second reader radiologist may ultimately lead to junior radiologists not having sufficient opportunities to review and interpret mammography images themselves, leading to a decline in competence over time and in turn affecting the quality of screening. Such deskilling may further worsen the identified problem of automation bias, which is consistent with previous studies on AI in healthcare [12, 15, 21, 22]. However, AI might introduce or reduce bias in decision-making, depending on whether explanations are transparent and align with clinical logic. Ethical concerns may arise as radiologists start to rely substantially on AI [23, 24]. Consequently, the concern of senior competence diminishing, and/or junior radiologists not obtaining the same competence as their senior colleagues, is a significant, long-term issue that needs to be considered. Thus, carefully following up on any tendency among individual radiologists to agree with the AI and to benchmark the radiologists’ performances, are important actions to reduce any automation bias [25]. In addition, Baltzer [25] argues the significance of radiologists receiving continuous training and education with the AI tool. This would not only clarify the strengths of AI, but also its limitations and consequently contribute to the radiologist making more informed decisions rather than automation-biased decisions [25].

The AI integration at Capio S:t Göran was identified as a risk with low likelihood but potentially severe impact, particularly in terms of data breaches or service disruption. Etges et al. [11] recognize that cybersecurity risks are the top enterprise risk for healthcare organizations. Since the AI server is located on-premises, it does not pose significant additional security risks. An on-premises solution allows organizations to have more control over their data, making them less vulnerable to data breaches and security threats compared to a cloud-based solution [26, 27]. However, while the integration of AI itself may not exacerbate cybersecurity risks at Capio S:t Göran hospital, it is still crucial to prioritize security measures, especially given the rising number of cyberattacks and the vulnerability of hospitals in general [28].

Technology acceptance plays a critical role in the successful integration of new technologies in organizations [29]. At Capio S:t Göran, the personnel involved in mammography screening have demonstrated a positive inclination toward the use of AI. It is however important to note that there were initial concerns among the personnel, which is a common reaction during organizational changes [13]. Furthermore, it is worth mentioning that, despite the personnel accepting the AI integration, radiographers expressed a sense of exclusion and a desire for more information. This highlights the importance of involving staff during organizational changes and acknowledging diverse perspectives [3, 17].

Partnerships with third-party entities in hospitals carry inherent risks, including lock-in effects, where the hospital becomes heavily dependent on the third party. This issue has been recognized by scholars in various industries [30–32]. However, for the breast imaging department at Capio S:t Göran, this risk was considered irrelevant due to the integration of the AI output into the regular PACS.

To our knowledge, some of the risks that our study discovered have not extensively been discussed in previous research. One such risk is the increased likelihood of a single radiologist overlooking clinical symptoms reported by radiographers when AI is implemented in clinical practice. While this finding is in line with previous work, our study uniquely frames it within a structured enterprise risk management perspective prior to AI deployment. As emphasized by Choi et al. [33], radiologists rely not only on mammogram findings but also on clinical symptoms provided by radiographers, which may not be visible on the mammograms but are crucial for accurate diagnosis.

Related to the above risk, an additional risk that warrants further investigation is the heightened pressure faced by first-reader radiologists who are aware that they are the sole responsible party for identifying any mistakes made by AI. Prioritizing the well-being of physicians is crucial in ensuring patient safety and delivering high-quality healthcare [11]. Considering that radiologists already experience substantial pressure due to heavy workloads, an increase in pressure could have adverse effects on their mental health and job performance.

The risk associated with setting the threshold value too high, determining the point at which abnormalities are considered significant, has not received sufficient research attention. Dembrower et al. (2023) [34] emphasize that a threshold value set too high increases the risk of undetected cancers. Conversely, setting it too low would likely increase false-positive findings and subsequently increase the workload. Striking the right balance is therefore crucial, necessitating further investigation to inform the development of national guidelines.

Radiographers face the challenge of addressing patient questions about the integration of AI, which has received limited attention in prior research. Therefore, it is crucial to implement educational programs for radiographers to equip them with the knowledge and skills necessary to provide accurate information and reassure patients effectively.

### Limitations

A qualitative single-case study design has allowed for in-depth inquiries. However, due to the study being delimited to one case, no statistical generalizations are claimed. Some of the risks and the risk mitigation actions identified might thus not apply to all healthcare settings. Secondly, this study has not evaluated the effectiveness of the suggested risk mitigation actions, which may result in uncertainty regarding their practical impact and efficacy of the actions. We acknowledge that our analysis did not formally distinguish between risk domains and risk sources, nor categorize mitigation strategies by response type (e.g., avoid, reduce, transfer). Future work could build on our findings by linking each risk to its underlying causes and evaluating response strategies accordingly. Additionally, it is crucial to acknowledge that there could be risks that we were not able to identify in this study. Involving more stakeholders could have contributed to a more comprehensive identification and assessment of risks. Inclusion of more senior management could have strengthened identification of strategic and financial risks This study explored risks perceived by professionals involved in the planned implementation of AI in screening mammography. The study did not include patient perspectives, which is a limitation of the current study but was the focus of a separate study from our group [35].

### Conclusions

To successfully introduce AI in clinical settings, healthcare organizations must establish proactive risk management and broaden their focus beyond patient safety. New risks may emerge post-integration and the efficacy of the proposed risk mitigation actions may diminish over time. It is therefore imperative to establish a standard practice of regularly monitoring both risks and mitigation actions. The engagement of diverse stakeholders is furthermore vital to ensure that a comprehensive approach is maintained when identifying and addressing risks.

The study’s identification of risks and mitigation actions could serve as a foundation for the development of best practices when implementing AI in clinical settings. The discussion on the risks as well as the measures that could be taken, regarding the use of AI in healthcare settings, is necessary to create and promote an ecosystem of trust, safety, and success.

## Supplementary Information

Additional file 1. The risk inventory including risk mitigation actions related to standalone AI for screening mammography. The risk inventory table shows 23 potential risks including 51 risk mitigation actions related to standalone AI for screening mammography.

## Data Availability

No datasets were generated or analysed during the current study.

## Abbreviations

AI: Artificial Intelligence
ASHRM: American Society for Healthcare Risk Management
ERM: Enterprise Risk Management
