# Supplementary material for: Risk inventory and mitigation actions for AI in medical imaging—a qualitative study of implementing standalone AI for screening mammography
Source: BMC Health Serv Res. 2025 Jul 30;25:998. doi: 10.1186/s12913-025-13176-9 (PMC12309172; doi:10.1186/s12913-025-13176-9)
Supplement: Supplementary file 1 — Additional file 1. The risk inventory including risk mitigation actions related to standalone AI for screening mammography. The risk inventory table shows 23 potential risks including 51 risk mitigation actions related to standalone AI for screening mammography. [file 12913_2025_13176_MOESM1_ESM.docx]

**Table S1.** Enumeration of risks and suggest risk mitigation actions, structured according to the risk domains provided by the enterprise risk management framework from the American Healthcare Risk Management Society.

| **Primary Risk Domain** | **Specific Risk** | **Description** | **Secondary Risk Domain(s)** | **Risk Mitigation Actions** |
| --- | --- | --- | --- | --- |
| Operational | Radiographers unable to answer difficult questions from patients about the AI implementation. | As patients become aware of AI, they will ask questions to the person they meet in the mammography suite - and that is the radiographer. | Strategic | - Education programs for radiographers to learn more about the AI implementation. - Brainstorm potential patient questions and provide radiologists and radiographers with suggested answers. - Inform patients regarding AI implementation through waiting-room signs. - Include specific agenda point in each workplace meeting ("APT") to follow-up radiographer experience and symptom reporting. |
|  | Workflow delays and disruptions caused by the AI vendor. | Lack of IT-support from the AI vendor or AI system downtime could lead to workflow delays and disruptions. | Patient Safety | - Automatic renewal of support agreement from the AI vendor in case the AI system goes end-of-life. - Ensure that reversion to human double-reading is easy. |
|  | IT department unable to deliver continuous AI availability. | When AI is used to read screening mammograms, delays by more than a few hours are not acceptable and should be rare in order not to disrupt the intended workflow | Financial  Technology | - Ensure the same service-level and safeguards as for other critical IT systems. - Retain the possibility to revert to a human-only workflow. |
| Patient Safety | Increased risk that patient-reported new lumps, breast symptoms or previous cancer noted by the radiographer is missed by the single radiologist when AI replaces a radiologist. | Radiologists take this information into account in making their decisions, but AI does not. Sometimes it may be indicative of cancer and should not be missed. The risk of missing might increase when only one radiologist instead of two review each exam. | Operational  Legal | - Check with the PACS provider if radiographer flagging lump, nipple discharge and retraction may steer the exam to a special list to heighten the radiologist attention. - New lumps (red dots) could be directly transferred to consensus discussion. - Remind the single radiologist to check radiographers' notes. - Educate nurses on which symptoms to note and not to note, e.g., new symptoms, not old ones. |
|  | AI performance is unknown for women with breast implants and other special cases. | The accuracy of the AI system in detecting cancer has not been validated for women with breast implants and other special cases which could lead to misdiagnosis. | Operational | - Ensure that women for which the AI was not validated in the clinical trial (ScreenTrust CAD) continue with human double-reading (plus AI). - Prioritize validation of AI performance for women with breast implants and others excluded from the clinical trial. |
|  | Setting the AI threshold value too high. | Setting the AI threshold value too high may increase the risk of cancers being missed as values below the threshold could still indicate cancer. | Legal | - Raise awareness of the need to clarify this question on a national and European level, e.g., include in the national care program how to calibrate the AI threshold (provide guidelines). |
|  | Automation bias. | The radiologist puts less trust in the judgment of themselves or their colleagues after viewing the AI information. Applicable to the consensus discussion (the preceding independent reading is blinded). | Operational  Human Capital  Legal | - Follow-up on each radiologist's tendency to agree with AI. - Follow-up the positive predictive value of consensus decisions: separate for AI flagged and radiologists flagged (as an indicator of automation bias or inverse automation bias). |
| Strategic | Patients might be resistant toward the use of AI. | There is a risk that patients may be less accepting of errors made by AI, compared to human radiologists, which could lead to reluctance to use of AI. |  | - Brainstorm potential patient questions and provide radiologists and radiographers with suggested answers. Continuously update the list of patient questions, discuss suggested answers. - Provide patients with written information about the AI implementation at the hospital. |
|  | Data safety issues in relation to the AI vendor.* | AI vendor having access to personal data could lead to increased vulnerability for the hospital. | Patient Safety  Technology | - Follow established IT security guidelines applicable to third-party IT system suppliers. |
|  | Costly to change to another AI vendor due to lock-in effects.* | The hospital might become reliant on the AI vendor, making it difficult to switch to alternative options. | Financial | - Ensure standardized interface to lower barriers to switch to another AI vendor. |
|  | Hospital management not seeing the value of the clinical AI implementation. | The hospitals decision makers do not recognize the benefits of implementing AI clinically. | Financial | - Present the existing research evidence to hospital management (identify benefits with the AI). - Conduct profitability assessment. |
| Financial | Price increase of AI system by vendor. | Long-term development of AI pricing is unclear. | Strategic | - Follow current procurement strategy as for other third parties. - Create mutual dependence (i.e., offer value to the AI vendor) so that AI supplier has incentives to keep prices low. |
|  | Repeated investments required later for the AI system. | AI systems have a limited lifespan which may require repeated investments over time. | Strategic | - Follow current IT investment strategy, including developing a long-term plan for future costs linked to technology investments. |
| Human Capital | Deteriorating competence of future radiologists. | The reduction in the number of exams that radiologists interpret could make it harder to train junior radiologists, and that it might be required that it is a senior radiologist that is the only human reader reducing the opportunities for junior radiologists to be in "live action". | Operational  Patient Safety | - Let junior radiologists make shadow assessments which do not affect the workflow senior radiologists during examination. - Let junior radiologist perform examination in a mode where the results are not considered in the actual assessment. - Design more effective training and evaluation systems to train juniors and to know when their performance have become adequately good. |
|  | Increased pressure on the single reader knowing they are the only radiologist to catch a mistake by AI. | The single reader, who is responsible for interpreting the exams may feel an increased pressure to catch any mistakes that may have been missed by the AI system. |  | - Assure radiologists that their performance is enough if they are senior or have passed the suggested screening accuracy assessment test. - Clarify to radiologists that AI has shown to be at least as good as a second radiologist. - Hold regular AI debriefings with radiologists to learn how they experience the AI-powered process. - Provide each radiologist with a list of the women they have declared healthy but are later recalled due to a suspicious finding. |
|  | Personnel not properly informed regarding the AI implementation. | Radiographers and radiologists need to understand how AI is implemented, what results are expected, and how to explain AI to colleagues and patients | Operational  Strategic | - Education programs and presentations for radiographers and radiologists to learn about the AI implementation. |
|  | The involved staff not accepting the AI implementation* | Worry among the involved radiologists and radiographers could affect the technology acceptance negatively. | Strategic | - Involve staff in the initial stages of AI implementation. - Contact other hospitals where AI has been implemented to take part of their experiences and receive reassurance. |
| Legal/Regulatory | Legal and regulatory uncertainties with AI. | Ensuring adherence to laws and regulations, such as the patient data act, MDR, GDPR, CE mark, and the AI Act can be time-consuming and complex. | Strategic  Financial | - Consult a medicolegal expert to understand if AI complies with current and upcoming laws and regulations. - Ensure that AI assessments are reimbursed on an equal level as radiologist assessments. |
|  | Liability uncertainties if AI misses a cancer. | There are currently no court cases demonstrating how the liability is distributed if AI misses a cancer. | Financial  Strategic | - Consult a medicolegal expert to understand the current responsibility and what could change with AI. - Clarify the responsibility for regular radiologists, the medically responsible radiologist, and the head of department. - Raise awareness of the need to clarify this question on a national and European level, e.g., include in national care program how to calibrate the AI threshold (provide guidelines). |
| Technology | The AI system malfunctions. | System errors and bugs could lead to AI system malfunction | Operational  Patient Safety | - Ensure adequate service level in the agreement with the AI vendor. - Ensure that the update and maintenance policy is adequate in the agreement with the AI vendor. |
|  | Increased cybersecurity risks when AI is implemented. * | If data is sent to, or stored in, cloud services it could lead to increased security risks | Operational  Patient Safety | - Ensure that the AI system follows established security protocols and determine if any additional security risks are introduced. |
|  | The performance of the AI algorithm might deteriorate. | The effects of changes, such as algorithm updates, new radiology equipment, or change of AI-vendor on the AI algorithm, are unknown. | Operational  Patient Safety | - Use external validation platform (e.g., VAI-B) or create an internal quality assurance dataset legalized through the decision by the Head of department. - Perform constancy-controls by regularly image physical phantoms with simulated lesions resulting in abnormality scores at various levels. - Perform frequent performance constancy follow up of the algorithm: - WEEKLY   - Prediction score distribution.   - Proportion of all exams that were flagged by AI.   - Proportion of recalled exams that were flagged by AI.   - Proportion of AI flagged exams that were recalled.   - Median AI scores: overall, flagged by radiologist, not flagged by radiologist, recalled. - MONTHLY   - Proportion of exams with cancer that were flagged by AI.   - Median AI scores of cancers. - The name and version number of each AI system should be stored in image meta data and linked to its output data. |
|  | Difficult for the radiologist to understand the reasoning behind AI’s output. | If the AI system is less user-friendly, it could impair the radiologist’s understanding and the trust in the AI-system’s output which negatively affect the delivery of care. | Patient Safety | - Make introductory presentations for new radiologists. - Ensure that the AI information is displayed on the images in a way that is easy to understand for radiologists. - Create PACS lists where radiologists can put exams with AI information they find puzzling – to be discussed in internal conference. |

*Not relevant to the AI implementation at Capio S:t Göran’s hospital.
